# Patient characteristics and pregnancy outcomes among Zika‐infected pregnant women: Epidemiologic surveillance data from two cities in Colombia, 2015–2016

**DOI:** 10.1002/ijgo.13041

**Published:** 2020-01-23

**Authors:** Jovana A. Ocampo Cañas, David Caviedes Combita, Helvert F. Molina Leon, Andrés M. Garcia Sierra, Luis J. Hernández Florez

**Affiliations:** ^1^ Public Health, Medical Education, and Professionalism Research group School of Medicine University of Los Andes Bogotá Colombia

**Keywords:** Colombia, Epidemiologic surveillance, Low birthweight, Pregnancy outcomes, Preterm birth, Zika virus

## Abstract

**Objectives:**

To describe the characteristics of pregnant women infected with Zika virus in two representative regions of Colombia, examine their pregnancy outcomes, and outline findings of the epidemiologic surveillance program established during the peak of the 2015–2016 epidemic.

**Methods:**

A cohort study conducted in the municipalities of Cali and Villavicencio using data from the National Public Health Surveillance System (SIVIGILA) and clinical follow‐up data from pregnant women. We describe sociodemographic characteristics, health insurance status, Zika virus, pregnancy‐related characteristics, and pregnancy outcomes.

**Results:**

A total of 1259 Zika‐infected pregnant women were identified in Cali; of these, 2.3% (n=27) experienced pregnancy loss, 9.5% (n=113) had preterm birth, and 7.9% (n=91) had a low birthweight neonate. In Villavicencio, 3.0% (n=13) experienced pregnancy loss, 6.9% (n=30) had preterm birth, and 6.7% (n=28) had a low birthweight neonate. Compared with the general population, this population of Zika‐infected pregnant women did not experience an increased frequency of preterm birth or low birthweight (relative risk of prematurity and low birthweight infant <1).

**Conclusions:**

Epidemiologic surveillance data showed that most neonates of Zika‐infected pregnant women were born at term, and that the frequency of low birthweight neonates was low. Good quality data were obtained from the surveillance registries.

## Abstract 2

Epidemiologic surveillance data showed that most newborns of Zika‐infected pregnant women were born at term, and that the frequency of low birthweight infants was low. Good quality data were obtained from the surveillance registries.

## 1 INTRODUCTION

The first Zika virus outbreak in Latin America was identified in Brazil in early 2015. During the period 2015–2016,^1^ the Brazilian epidemiologic surveillance system reported a significant increase in the incidence of microcephaly among newborns—from 0.5 cases per 10 000 live births in September 2015 to 20 cases per 10 000 live births in November 2015.^2^ Subsequent studies concluded that a causal relationship existed between Zika virus infection and microcephaly.^3^ This conclusion was based on the temporal relationship between exposure to the vector during pregnancy and subsequent microcephaly in the fetus, which occurred among residents of endemic areas^4^, ^5^ as well as among travelers.^6^ Viral genetic material was also found^7^ and Zika virus was isolated in the cerebral tissue of fetuses with microcephaly.^8^

Due to the reported increase in cases of microcephaly, the governments of countries affected by the epidemic strengthened their surveillance systems for birth defects and issued alerts with recommendations to delay pregnancies where possible^9^ and to provide close follow‐up for high‐risk pregnant women. In Colombia, the conditions for propagation and transmission of Zika virus are excellent since the vector is found in urban and rural areas below 2200 m of altitude.^1^

In Colombia, epidemiologic surveillance of Zika virus officially started in August 2015. Epidemiologic alerts were generated, and protocols and management guidelines were designed. These were implemented in each of the departments and municipalities, which allowed the epidemic to be monitored throughout the national territory. Between April 2015 and August 2016, 11 944 pregnant women were reported to have Zika virus in Colombia, of which 12.4% (n=1484) were diagnosed positive using reverse transcriptase polymerase chain reaction (RT‐PCR).^1^

Zika virus cases were reported throughout the country. Cities such as Cali and Villavicencio reported high numbers of cases of vector‐transmitted diseases due to their location and altitude. Cali is the capital of Valle del Cauca Department, southwest of Bogotá; Villavicencio is the capital of Meta Department, southeast of Bogotá. Although these cities did not report the greatest incidence of Zika cases, they did have solid epidemiologic surveillance systems in place that allowed officials to track Zika‐related and other outcomes, including preterm birth and low birthweight.

The aim of the present study was to describe the population of Zika‐infected pregnant women from Cali and Villavicencio and their pregnancy outcomes (particularly preterm birth and low birthweight newborns). A secondary objective was to describe the epidemiologic surveillance system in these two Colombian cities during the 2015–2016 epidemic.

## 2 MATERIALS AND METHODS

In Colombia, local Zika virus transmission was confirmed in October 2015. At that time, systematic reporting of cases of suspected Zika virus was initiated through the National Public Health Surveillance System (SIVIGILA) of the National Institute of Health. This information is compiled at the municipal level and then at the regional and national level. Because pregnant women are considered a high‐risk population, suspected cases were confirmed with Zika virus RT‐PCR and cases were followed to determine pregnancy outcomes.

The present study analyzed a cohort of Zika‐infected pregnant women living in Cali and Villavicencio who were diagnosed during the epidemic period from October 11, 2015 to July 16, 2016. The principal source of information was the SIVIGILA national registry of reported Zika virus cases. From these, pregnant women who had been registered as infected with the virus were selected and examined. This information was complemented by the epidemiologic surveillance systems in each city to ensure that no cases were missed as data were transmitted from regional to national level.

We completed a descriptive analysis of the sociodemographic characteristics of the infected pregnant women, their pregnancy‐related care patterns, and pregnancy outcomes. Using the symptom dates reported, we estimated the trimester of pregnancy during which women were infected (infection date=date of symptom onset − 15 days of incubation period). We conducted bivariate analyses to evaluate the relationship between the timing of Zika virus infection and type of institution in which the woman received prenatal and delivery care (public or private) and pregnancy outcomes such as preterm birth (less than 37 weeks) and low birthweight (less than 2500 g).

Using the date of symptom onset, we calculated a probable date of infection. We then conducted bivariate analyses to look for patterns related to prematurity and low birthweight among different subgroups (first, second, or third trimester at the time of infection). We conducted the same analysis using the type of healthcare institution attended for prenatal care as the exposure.

We calculated the relative risk of low birthweight and prematurity by comparing the rate in the population of Zika‐infected women with that in the general population. This measurement estimates the risk of these two adverse pregnancy outcomes in the population of Zika‐infected pregnant women and compares it with the expected frequency in the general population. We conducted all data analyses using R software version 2.5.1.^10^

This study was approved by the Ethics Committees of the University of Los Andes (certificate No. 658, 2016) and the Pan‐American Health Organization (certificate No. OPS‐2017‐04‐0042).

## 3 RESULTS

A total of 1758 pregnant women with Zika virus were identified in SIVIGILA, of which 71.6% (n=1259) were from Cali. Most women were young, with a mean age of 26 years for both cities (Table 1). In the city of Cali more women were Afro‐American (n=27, 2.2%) compared with other non‐mestizo ethnic groups (<1% were of indigenous or gypsy ethnicity in both cities).

**Table 1:** Clinical and epidemiologic characteristics of Zika‐infected pregnant women (n=1758) reported to SIVIGILA between October 2015 and July 2016.^a^

Characteristics	Cali (n=1259)	Villavicencio (n=499)
Age, years		
Mean	26.8 ± 5.9	26.1 ± 6.0
Range	12–46	14–43
Ethnicity	(n=1257)	(n=440)
Indigenous	4 (0.3)	1 (0.2)
Afro‐Colombian	27 (2.2)	0 (0)
Gypsy	5 (0.4)	5 (1.1)
Other groups	1221 (97.1)	434 (98.6)
Type of social security regimen	(n=1258)	(n=440)
Contributive (employer‐based)	979 (77.9)	279 (63.4)
Subsidized	247 (19.7)	113 (25.7)
Other/undetermined	15 (1.1)	39 (8.9)
None	17 (1.4)	9 (2.0)
Interval between symptom onset and presentation, days	(n=1257)	(n=440)
Mean	3.2 ± 8.4	3.1 ± 8.6
Range	0–153	0–147
Gestational age at time of symptom onset, weeks	(n=1251)	(n=445)
Mean	21.2 ± 9.6	21.7 ± 9.7
Range^b^	0–40	0–40
Trimester at time of symptom onset	(n=1251)	(n=445)
First trimester	319 (24.5)	100 (22.5)
Second trimester	565 (45.2)	201 (45.2)
Third trimester	367 (29.3)	144 (32.3)

a Values are given as mean ± SD, range, or number (percentage).

b Gestational age was calculated as number of pregnancy weeks completed. For example, week 0 corresponds to the period from day 1 to day 6 of pregnancy.

The majority of women received healthcare services paid for by employer‐based insurance plans (contributive regimen): 77.9% (n=979) in Cali and 63.4% (n=279) in Villavicencio. The remaining pregnant women had subsidized insurance through the public health sector; very few had private insurance that was not employer‐based insurance.

On average, pregnant women sought medical care a few days after symptom onset: 3.2 ± 8.4 days in Cali and 3.1 ± 8.6 days in Villavicencio. In both cities the majority of women were in the second trimester at symptom onset: 45.2% (n=565) in Cali and 45.2% (n=201) in Villavicencio (Table 1).

Mean gestational age at delivery was 37.9 ± 3.9 weeks in Cali and 38.1 ± 5.0 weeks in Villavicencio. The proportion of pregnancies that ended in preterm birth was 9.5% (n=113) in Cali and 6.9% (n=30) in Villavicencio. The rate of pregnancy loss was 2.3% (n=27) in Cali and 3.0% (n=13) in Villavicencio. Most of these were losses prior to 22 weeks of pregnancy: 1.3% (n=160 in Cali and 2.3% (n=10) in Villavicencio. The rate of neonatal death was 0.4% (n=5) in Cali and 0.2% (n=1) in Villavicencio (Table 2).

**Table 2:** Pregnancy outcomes for Zika‐infected pregnant women (n=1758) reported to SIVIGILA between October 2015 and July 2016.^a^

Pregnancy outcomes	Cali (n=1259)	Villavicencio (n=499)
Gestational age at delivery, weeks	(n=1187)	(n=431)
Mean	37.9 ± 3.9	38.1 ± 5.1
Range	4–41	3–41
*Pregnancy outcomes*	(n=1195)	(n=435)
Data available for gestational age at delivery	1168 (97.7)	422 (97.0)
Term delivery (≥37 wk)	1055 (88.3)	392 (90.1)
Preterm (<37 wk)	113 (9.5)	30 (6.9)
Pregnancy loss	27 (2.3)	13 (3.0)
<22 wk	16 (1.3)	10 (2.3)
>22 wk (prior to delivery)	11 (0.9)	3 (0.7)
Neonatal death	5 (0.4)	1 (0.2)
Early neonatal death^b^ (0–7 days)	4 (0.3)	1 (0.2)
Delayed neonatal death^b^ (7–30 days)	1 (0.1)	0 (0)
Data available for birthweight	(n=1159)	(n=421)
Low birthweight	91 (7.9)	28 (6.7)
Adequate birthweight	1068 (92.2)	393 (94.4)
Relative risk of low birthweight (95% CI)^c^	0.80 (0.65–0.98)	1.01 (0.68–1.46)
Relative risk of prematurity (95% CI)^d^	0.43 (0.37–0.53)	0.51 (0.37–0.74)

a Values are given as mean ± SD, range, or number (percentage) unless otherwise indicated.

b All the cases of neonatal deaths occurred among liveborn premature infants.

c Calculated as the proportion of low birthweight neonates among Zika‐infected pregnant women over the proportion of low birthweight neonates in the general population in the city during the same time period.

d Calculated as the proportion of premature neonates among Zika‐infected pregnant women over the proportion of premature neonates in the general population in the city during the same time period.

The relative risk of both low birthweight and prematurity was less than 1 in Cali. In Villavicencio, the relative risk of low birthweight was equal to 1 and that of prematurity was less than 1 (Table 2).

In Cali, the frequency of prematurity among pregnant women who contracted Zika in the first and second trimester was 10.6% (n=329) and 10.5% (n=531), respectively. In Villavicencio these rates were 8.6% (n=105) and 8.9% (n=213), respectively. Among women who contracted the virus in the third trimester, the rate of prematurity was 7.3% (n=303) and that of low birthweight was 6.3% (n=303) in Cali, while in Villavicencio it was 1.9% (n=104) for both prematurity and low birthweight when contracted in the third trimester (Table 3).

**Table 3:** Bivariate analysis of trimester at infection and type of healthcare institution and pregnancy outcome for liveborn neonates born to Zika‐infected women between October 2015 and July 2016.^a^

Variable	Cali			Villavicencio		
	Total	Preterm	Low birthweight	Total	Preterm	Low birthweight
Trimester at Zika infection						
First trimester	n=329	35 (10.6)	27 (8.4)	n=105	9 (8.6)	7 (6.7)
Second trimester	n=531	56 (10.5)	45 (8.5)	n=213	19 (8.9)	19 (8.9)
Third trimester	n=303	22 (7.3)	19 (6.3)	n=104	2 (1.9)	2 (1.9)
Type of healthcare institution						
Public	n=184	18 (9.8)	15 (8.2)	n=114	12 (10.5)	11 (9.7)
Private	n=984	95 (9.7)	76 (7.8)	n=307	18 (5.9)	17 (5.5)

a Values are given as number (percentage) unless otherwise indicated.

Most reported cases of Zika originated from private healthcare institutions: 84.3% (n=1061) in Cali and 72.1% (n=323) in Villavicencio. On average, private institutions in Cali took 1.5 ± 9.7 days to report cases to the epidemiologic surveillance system, while those in Villavicencio took 0.5 ± 2.8 days. In the public sector, the average interval from presentation to case report was 2.2 ± 10.4 days in Cali and 1.6 ± 5.4 days in Villavicencio (Table 4).

**Table 4:** Epidemiologic surveillance data for pregnant women infected with Zika virus between October 2015 and July 2016.^a^

Characteristics	Cali	Villavicencio
Type of healthcare institution that reported the case	(n=1259)	(n=448)
Public	198 (15.7)	125 (27.9)
Private	1061 (84.3)	323 (72.1)
Time from health visit to SIVIGILA report, days	(n=1257)	(n=440)
Overall	1.6 ± 9.8	0.9 ± 3.8
Public healthcare institution	2.2 ± 10.4	1.6 ± 5.4
Private healthcare institution	1.5 ± 9.7	0.5 ± 2.8
Case classification	(n=1259)	(n=449)
Laboratory confirmed	248 (19.7)	245 (54.6)
Clinically confirmed	992 (78.8)	204 (45.4)
Suspected	19 (1.5)	0 (0)
Variables with missing data	(n=1259)	(n=449)
Final maternal outcome	64 (5.1)	14 (3.1)
Final fetal outcome	64 (5.1)	14 (3.1)
Case classification	0 (0)	0 (0)
Healthcare institution that reported the case	0 (0)	1 (0.2)
Date of symptom onset	0 (0)	0 (0)
Date of healthcare visit	2 (0.2)	9 (2.0)
Date of case report	2 (0.2)	9 (2.0)
Last menstrual period	6 (0.5)	4 (0.9)
Date of pregnancy end	72 (5.7)	16 (3.7)
Birthweight	100 (7.9)	28 (6.2)
Week of pregnancy at end of pregnancy	79 (6.3)	19 (4.2)

Abbreviation: SIVIGILA, National Public Health Surveillance System.

a Values are given as mean ± SD or number (percentage).

It is worth noting that in Cali 78.8% (n=992) of cases were diagnosed clinically and only 19.7% (n=248) were confirmed by laboratory testing. In contrast, in Villavicencio, 54.6% (n=245) were laboratory confirmed. An evaluation of the data for completeness revealed that pregnancy outcome was not recorded for 5.1% (n=64) of cases in Cali and 3.1% (n=14) of cases in Villavicencio. Information was missing on date or trimester of delivery in 6.3% (n=79) of cases in Cali and 4.2% (n=19) of cases in Villavicencio, and on birthweight in 7.9% (n=100) of cases in Cali and 6.2% (n=28) of cases in Villavicencio (Table 4).

## 4 DISCUSSION

Demographic characteristics of Zika‐infected women were similar in the two municipalities in this study. Most women had employer‐based insurance, which is generally a marker of middle‐class status. This is not the usual pattern for vector‐borne diseases, which usually disproportionately affect populations of low socioeconomic status.^11^ Similarly, the proportion of Afro‐Colombian women affected by Zika virus in Cali was lower than the proportion of Afro‐Colombian women in the municipality, which has a larger Afro‐Colombian population than the rest of the country. Some epidemiologic studies of vector‐borne diseases and arboviruses have identified racial differences in susceptibility to infection and severity of illness.^12^, ^13^

The pattern we observed could be due to under‐reporting of cases among the poorest population who attend public healthcare institutions. Another possibility is that this population may not seek healthcare services owing to sociocultural factors or barriers to accessing health care. Other hypotheses include immunity to Zika virus due to frequent infections such as dengue, or other protective factors such as ethnicity or even pregnancy.^14^

In this study we observed a latency of more than 3 days from symptom onset to first medical consultation, which suggests that educational and communication strategies were not very effective, or that there are barriers to accessing care. According to the government's control strategies during the epidemic, any pregnant woman with signs or symptoms suggestive of Zika virus was to seek care immediately, which would facilitate the collection of samples for laboratory diagnosis.^15^ Some studies have reported a low frequency of symptoms among infected individuals, or very mild symptoms that could explain the delay in seeking medical attention.^16^

The frequencies of prematurity and low birthweight in these populations were lower than those of the general population in the same municipalities, as demonstrated by calculating relative risk. There was also no difference in terms of pregnancy outcomes according to trimester at infection.

Some observational studies have reported an increased frequency of intrauterine growth restriction and low birthweight in neonates of pregnant women with a history of Zika virus^16^; however, other studies showed minimal impact of Zika infection on fetal growth.^5^, ^7^

Unfortunately, access to data on the occurrence of congenital malformation was restricted; we were therefore unable to include these data in this analysis, but we hope that it will be published in additional studies conducted by other investigators in the country.^17^, ^18^ Our focus was on observing pregnancy outcomes and on describing the quality of demographic surveillance system data.

The patterns of variables in our study were similar in both cities. The frequency of missing data was low, which suggests that the quality of individual registries was high.

This study shows that more research is needed about Zika virus during pregnancy but also about the impact of social and cultural contexts and of health system response strategies on clinical outcomes. Describing epidemiologic surveillance systems for Zika virus during pregnancy will help optimize case detection and improve adherence to diagnostic and follow‐up protocols, which in turn will increase knowledge about the disease.

## AUTHOR CONTRIBUTIONS

JAOC and HFM designed the study. JAOC, DC, AMG, and LJH collected the data. JAOC, HFM, AMG, and DC contributed to data analysis and interpretation, as well as with manuscript preparation. The authors are responsible for the opinions expressed in this publication, which do not necessarily reflect the opinions or political decisions of the institutions to which they are affiliated, or of the institutions who kindly supported the study.

## CONFLICTS OF INTEREST

The authors have no conflicts of interest.
